# Design, implementation and evaluation of a national campaign to deliver 18 million free long-lasting insecticidal nets to uncovered sleeping spaces in Tanzania

**DOI:** 10.1186/1475-2875-12-85

**Published:** 2013-03-04

**Authors:** Sabine Renggli, Renata Mandike, Karen Kramer, Faith Patrick, Nick J Brown, Peter D McElroy, Wilhelmina Rimisho, Amina Msengwa, Ally Mnzava, Rose Nathan, Romanus Mtung’e, Rita Mgullo, Jane Lweikiza, Christian Lengeler

**Affiliations:** 1 Swiss Tropical and Public Health Institute, P.O. Box, 4002, Basel, Switzerland; 2 University of Basel, Basel, Switzerland; 3 National Malaria Control Programme, Ministry of Health and Social Welfare, Dar es Salaam, Tanzania; 4 Mennonite Economic Development Associates Tanzania, Dar es Salaam, Tanzania; 5 US President’s Malaria Initiative, Centers for Disease Control and Prevention-Tanzania, Dar es Salaam, Tanzania; 6 University of Dar es Salaam, Dar es Salaam, Tanzania; 7 Ifakara Health Institute, Dar es Salaam, Tanzania; 8 Population Services International, Dar es Salaam, Tanzania; 9 World Vision Tanzania, Dar es Salaam, Tanzania; 10 Tanzania Red Cross Society, Dar es Salaam, Tanzania

**Keywords:** Malaria, Vector control, Insecticide-treated nets, Long-lasting insecticidal nets, Distribution campaign, Tanzania

## Abstract

**Background:**

Since 2004, the Tanzanian National Voucher Scheme has increased availability and accessibility of insecticide-treated nets (ITNs) to pregnant women and infants by subsidizing the cost of nets purchased. From 2008 to 2010, a mass distribution campaign delivered nine million long-lasting insecticidal nets (LLINs) free-of-charge to children under-five years of age in Tanzania mainland. In 2010 and 2011, a Universal Coverage Campaign (UCC) led by the Ministry of Health and Social Welfare (MoHSW) was implemented to cover all sleeping spaces not yet reached through previous initiatives.

**Methods:**

The UCC was coordinated through a unit within the National Malaria Control Programme. Partners were contracted by the MoHSW to implement different activities in collaboration with local government authorities. Volunteers registered the number of uncovered sleeping spaces in every household in the country. On this basis, LLINs were ordered and delivered to village level, where they were issued over a three-day period in each zone (three regions). Household surveys were conducted in seven districts immediately after the campaign to assess net ownership and use.

**Results:**

The UCC was chiefly financed by the Global Fund to Fight AIDS, Tuberculosis and Malaria with important contributions from the US President’s Malaria Initiative. A total of 18.2 million LLINs were delivered at an average cost of USD 5.30 per LLIN. Overall, 83% of the expenses were used for LLIN procurement and delivery and 17% for campaign associated activities. Preliminary results of the latest Tanzania HIV Malaria Indicator Survey (2011–12) show that household ownership of at least one ITN increased to 91.5%. ITN use, among children under-five years of age, improved to 72.7% after the campaign. ITN ownership and use data post-campaign indicated high equity across wealth quintiles.

**Conclusion:**

Close collaboration among the MoHSW, donors, contracted partners, local government authorities and volunteers made it possible to carry out one of the largest LLIN distribution campaigns conducted in Africa to date. Through the strong increase of ITN use, the recent activities of the national ITN programme will likely result in further decline in child mortality rates in Tanzania, helping to achieve Millennium Development Goals 4 and 6.

## Background

The year 2010 was the deadline set by the Roll Back Malaria (RBM) Partnership to reach universal coverage for all populations at risk with locally appropriate malaria interventions, as well as to reduce global malaria cases and deaths from 2000 levels by 50% [1]. This was to be accomplished through the scale up of core malaria control interventions, such as the use of insecticide-treated nets (ITNs), indoor residual spraying, intermitted preventive treatment for pregnant women and artemisinin-based combination therapy [1]. These activities were expected to contribute to the achievement of the malaria-specific Millennium Development Goal (MDG) 6 by 2015 [1]. Given that malaria accounted in 2008 for 16% of deaths in children under-five years of age in Africa, reduction in malaria is also critical for achieving MDG 4 [2-4].

ITNs are a very effective measure for malaria control and high use reduces the incidence of symptomatic malaria episodes by 50% and can lower rates of all-cause mortality up to 29% [5]. In Tanzania mainland the responsibility of sustainably and equitably scaling up ITN use lies with the Ministry of Health and Social Welfare (MoHSW). Therefore, the National Insecticide Treated Nets (NATNETS) Programme was established in 2000 under the National Malaria Control Programme (NMCP) of the MoHSW [6,7]. As of late 2012, the programme has worked with numerous partners and been financed by a range of bilateral and multilateral donors. The ITN Cell, a unit within NMCP, coordinates and facilitates all NATNETS activities. It is funded by the Swiss Agency for Development and Cooperation (SDC) through the NETCELL Project, which is implemented by the Swiss Tropical and Public Health Institute in Basel and provides staff and technical support to the unit [8,9].

One of the ITN distribution mechanisms implemented by the NATNETS Programme is the Tanzanian National Voucher Scheme (TNVS). The TNVS has made ITNs widely available and accessible to pregnant women (since 2004) and infants (since 2006) through a voucher system that subsidizes the cost of nets purchased in commercial retail outlets [10]. However, in 2007/8 the MoHSW and other stakeholders considered the rate of increase in ITN ownership and use through the TNVS as too low and inequitable to reach RBM targets of universal coverage by 2010 [11,12]. As a result, the policy to distribute nets at no cost to beneficiaries through mass campaigns was adopted by the NATNETS partners to complement the TNVS. Between August 2008 and May 2010 the Under-five Catch-up Campaign (U5CC) delivered about nine million long-lasting insecticidal nets (LLINs) free-of-charge to every child under the age of five years across Tanzania mainland [11]. Additionally, a plan was developed by NMCP and NATNETS partners in 2008 to implement a second campaign, called the Universal Coverage Campaign (UCC). The aim of the UCC was to issue free LLINs to all sleeping spaces not yet covered through the TNVS and the U5CC. Following receipt of funds from GFATM in late 2009 and the procurement of LLINs during the first half of 2010, implementation of the UCC commenced in July 2010 and was completed in October 2011. This report summarizes the design, implementation and evaluation of the UCC. The financial cost of the campaign and coverage data post-campaign are also presented.

## Methods

**Figure 1:**
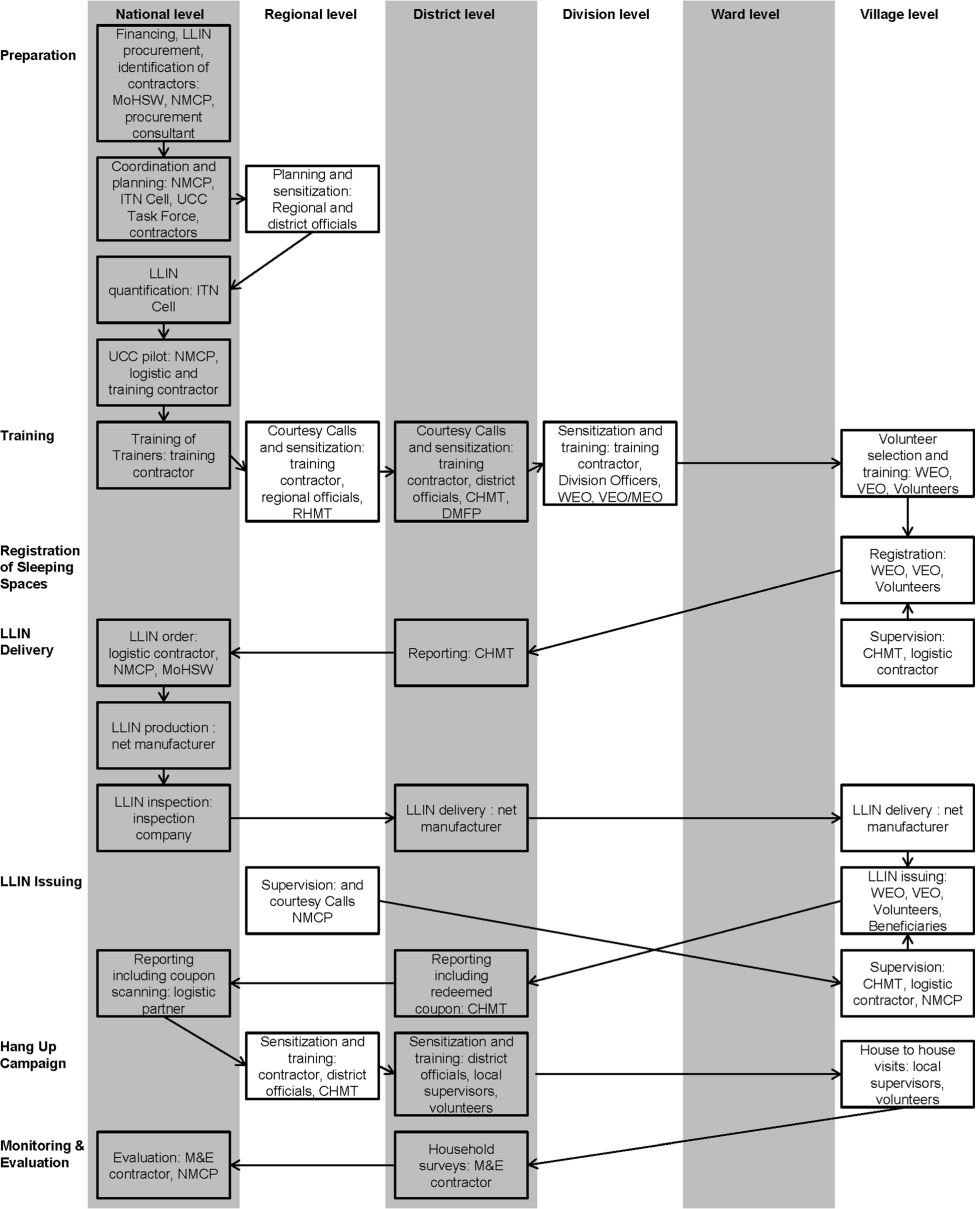
**Sequence of Universal Coverage Campaign activities at different government administration levels**, **mainland Tanzania.** NMCP = National Malaria Control Programme; ITN = insecticide-treated net; UCC = Universal Coverage Campaign; LLIN = long-lasting insecticidal net; MoHSW = Ministry of Health and Social Welfare; M&E = Monitoring and Evaluation; RHMT = Regional Health Management Team; DMFP = District Malaria Focal Person; WEO = Ward Executive Officer; VEO/MEO = Village/Street Executive Officer; CHMT = Council Health Management Team.

The UCC was designed, implemented and evaluated in multiple, phased steps described below in chronological order (Figure 1).

### Preparation

#### Financing, LLIN procurement and identification of contractors

In July 2008, Tanzania’s Country Coordinating Mechanism submitted a Round 8 proposal to GFATM. The grant agreement between the Government of Tanzania and the GFATM was signed in September 2009. The aim of the UCC was to distribute free LLINs to all the sleeping spaces that had not been provided with a net through previous NATNETS activities and to achieve at least 80% net usage, defined as universal LLIN coverage [1].

An open and competitive international tender to supply and deliver LLINs to village level was issued in March 2010 by the procurement consultant (Mennonite Economic Development Associates (MEDA)) on behalf of MoHSW. The tender was awarded to the most competitive bidder, A-Z Textile Mills Ltd, manufacturer of the Olyset™ net under license from Sumitomo Chemical Co.

The grant sub-recipients had already been identified by the Country Coordinating Mechanism during the development of the GFATM Round 1 Rolling Continuation Channel (RCC) proposal and as the two grants (RCC and Round 8) ran in parallel, the Country Coordinating Mechanism endorsed the utilization of the same sub-recipients. After signature of the grant agreement, sub-recipients were contracted by the MoHSW through its Procurement Management Unit. Contractors and their role are given in Table 1.

**Table 1:** Contractors and their role

**Contractor**	**Role**
Mennonite Economic Development Associates	(1) Provision of consultancy services for LLIN procurement, (2) managing the logistics and (3) redistribution of surplus stocks
World Vision Tanzania	Training at national, regional, district and division level
Population Services International	Social mobilization
KPMG	Financial and procedural audits
Ifakara Health Institute	Monitoring and evaluation
Tanzania Red Cross Society *	LLIN hang-up campaign

*Tanzania Red Cross Society has been separately funded and contracted by PMI.

#### Coordination and planning

ITN Cell personnel together with other NMCP staff and in collaboration with the UCC contractors coordinated the planning and implementation of the nationwide campaign. Regular NATNETS steering committee and coordination meetings [6] were responsible respectively for overseeing and coordinating the UCC. Additionally, regular UCC task force meetings were held to ensure that UCC plans were followed and designated activities were organized, implemented and monitored.

At regional level, the campaign planning process began with a series of meetings with the regional and district officials. The purpose of these meetings was to fully inform and consult these officials with regard to effective LLIN delivery schedules and communication strategies as well as the training of local government stakeholders based on the demographic and geographic characteristics of each district.

**Figure 2:**
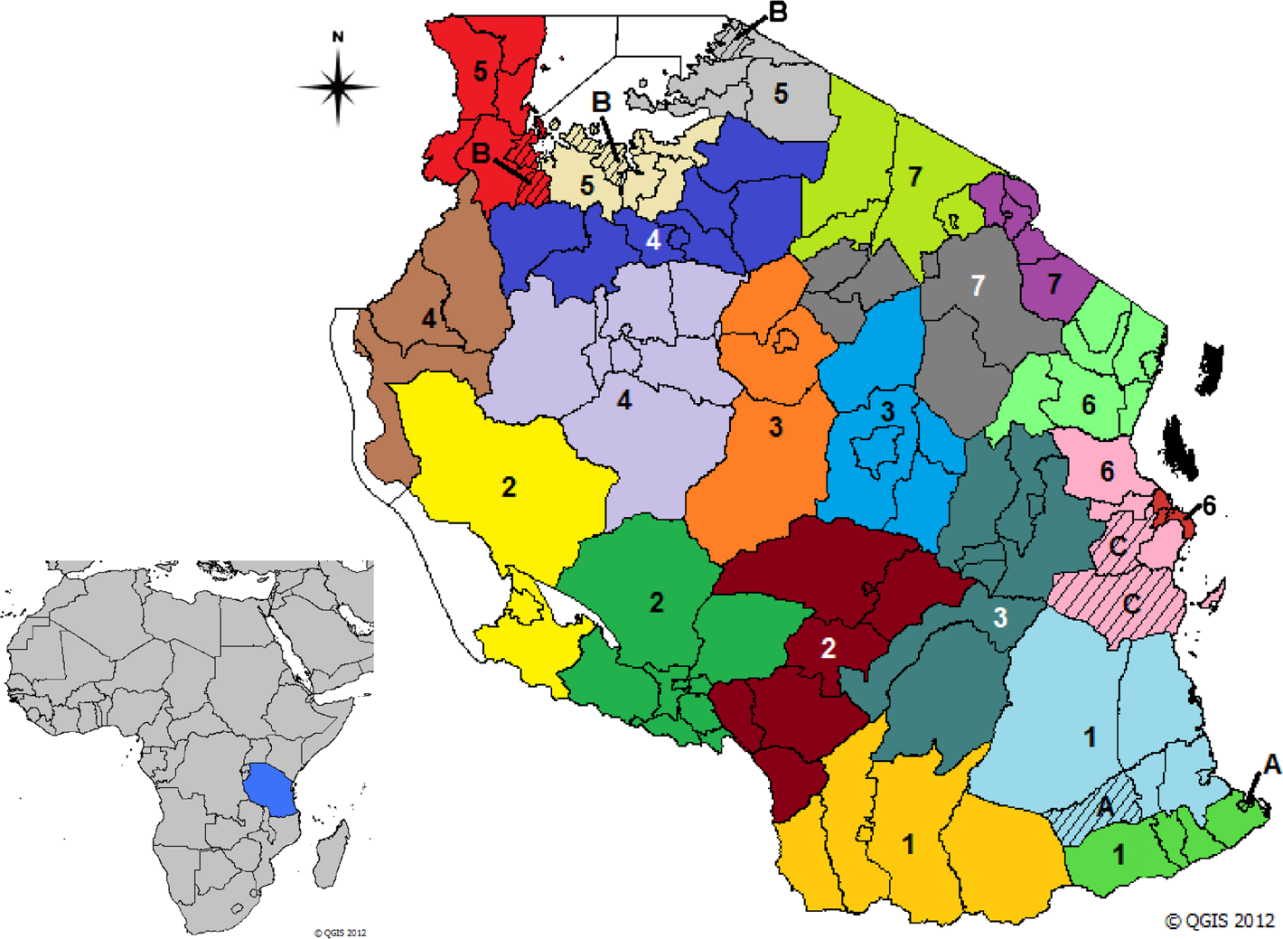
**Map of the United Republic of Tanzania.** Zones (consisting each of three coloured administrative regions) are numbered according to their implementation order. 1: Southern, 2: Southern Highlands, 3: Central, 4: West Lake, 5: Lake, 6: Coastal, 7: Northern. Shaded areas represent districts within regions in which household evaluation surveys were conducted. **A**: Southern zone (Nachingwea and Mtwara Urban Districts; **B**: Lake zone (Sengerema, Rorya and Chato Districts); C: Coastal zone (Kisarawe and Rufij Districts).

**Figure 3:**
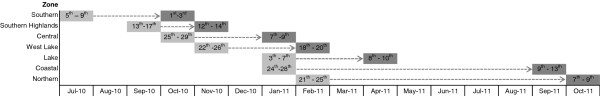
**Universal Coverage Campaign implementation timetable**, **mainland Tanzania.** The Universal Coverage Campaign was implemented on a rolling basis from one zone to another. Light grey indicates the household registration dates and dark grey the LLIN issuing dates in the corresponding zones.

To ensure optimal coordination and effective implementation, the 21 regions of mainland Tanzania were divided into seven zones: Southern, Southern Highlands, Central, West Lake, Lake, Coastal and Northern Zones (Figure 2). UCC activities started in June 2010 and proceeded on a rolling basis from one zone to another until completion in October 2011 (Figure 3).

#### LLIN quantification

The ITN Cell was responsible for the quantification of the LLIN requirements. One planning shortfall was that this exercise was conducted before the U5CC had begun and its lessons could be learned [11]. Thus, the number of required nets was derived from the National Bureau of Statistics projections for 2010 based on the 2002 Tanzanian National Census and the 2004 Demographic and Health Survey (DHS) data (Table 2).

**Table 2:** **Quantification of the long**-**lasting insecticidal nets requirements**, **mainland Tanzania**

**Item**	**Value**	**Source**
2010 Total Population Projection	41,914,311	Source: NBS National Projections Volume X11 2006 Table 11 p. 168
Average Household Size	4.8	Source: Table 2.2 DHS 2004
Number of Households	8,732,148	Calculated from above data
Number of sleeping spaces @ 2.5 sleeping spaces per household	21,830,370	Estimate. To be confirmed during U5 Registration process in 2008/9
Number of sleeping spaces to be covered in 2008/9 Under-five Catch-up Campaign	7,222,171	Source: NBS National Projections Volume X11 2006. Table 11 p. 167
Uncovered sleeping spaces covered in second campaign	14,608,199	Calculated from above data
Average number of people protected by each LLIN distributed by both campaigns	1.92	Calculated from above data

Source: Original Procurement and Supply Management Plan, Round 8, Global Fund to Fight AIDS, Tuberculosis and Malaria (unpublished document, 2008), Ministry of Finance and Economic Affairs, United Republic of Tanzania: Original Procurement and Supply Management Plan (unpublished document, 2008). *NBS* = National Bureau of Statistics; *DHS* = Demographic and Health Surveys.

#### UCC pilot

While awaiting the launch of the UCC, the logistics and training contractors along with NMCP implemented a pilot distribution to test and evaluate the effectiveness of the training materials and procedures. It was aimed at gaining field experience and building recommendations for future UCC planning and design. The pilot was conducted in three villages of the Ilemela District in Mwanza Region during 20 days in January 2010. The pilot resulted in a number of specific recommendations to improve training sessions and program design, which were incorporated into the main campaign (MEDA Tanzania: UCC Mwanza Pilot, Final Report. unpublished).

### Training

#### Regional level

Prior to initiating training activities at the regional level, field staff of the training contractor attended a Training of Trainers workshop to become familiar with the curriculum and training materials. Afterwards, at regional level, these field staff visited the Regional Medical Officer and the Regional Commissioner to brief them about the UCC and the required training and promotion activities to be held in their region. A sensitization meeting with each Regional Health Management Team was also held to discuss the UCC implementation.

#### District level

Similar activities were conducted at district level. The District Medical Officer (DMO) and the District Executive Director were informed about the UCC and the need to train local government officials. A one-day meeting was organized with Council Health Management Teams (CHMTs) in each district to explain the UCC. Additionally, the training contractor organized orientation for District Malaria Focal Persons (DMFPs), who teamed up with the trainers for the training of local government officials.

#### Division level

Local government officials such as Division Secretaries and Ward and Village Executive Officers (WEO and VEO) were instructed about the UCC in general and provided with the procedures regarding registration and issuing. They were informed about their responsibilities for supervising and reporting the whole process as well as for sensitizing the community. VEOs were also trained on how to select and train volunteers to perform the household sleeping space registration and LLIN issuing processes in each village (World Vision Tanzania: Universal Coverage Campaign, Final Implementation Report, unpublished).

### Registration of sleeping spaces

Registration took place in all 14,255 villages in mainland Tanzania. The exercise was done in one zone (three regions) at a time. At village level, VEOs selected and trained four literate and respected community members as volunteers. During a five-day process volunteers conducted house-to-house registration under the supervision and coordination of the VEOs and WEOs. Upon arrival at each household the volunteers used universal coverage registration cards (UCRC) booklets, consisting of sequentially numbered and bar-coded coupons and their corresponding carbon copies, to record all sleeping spaces not yet covered through the TNVS and the U5CC. A sleeping space was defined as any bed, sleeping mat or floor space that could be potentially covered by a net. Afterwards, the head of the household was issued one UCRC coupon for each eligible sleeping space and was instructed to bring the coupon to the assigned LLIN issuing point on one of the three designated issuing days. Households with no one at home received a sticker on the door requesting the household to visit the VEO’s office for registration. After the registration, all UCRC booklets were collected from the volunteers and used by the VEO to complete the Village Registration Report. The WEO then compiled all Village Registration Reports into a Ward Registration Report.

The CHMT then collected the Ward Registration Reports and paid allowances to the volunteers and the local government officials who participated in the registration activities. The reports were submitted to the logistics contractor who compiled all data into district packing lists. The logistics contractor also supervised the registration process together with CHMT members to ensure that procedures were correctly followed.

### Buffer stock management and LLIN delivery

#### Buffer stocks management

Based on the experience from the U5CC, the MoHSW decided to maintain two different buffer stocks of LLINs. The buffer stock at village level was an additional 5% over the number of registered sleeping spaces. By rounding the actual number of nets needed up to the nearest 40 (the number of nets in a single bale), another 2% on average was automatically added to the village buffer stock. At district level, and under the control of the DMO, an additional 23% of the total number of registered sleeping spaces in the district was used as buffer stocks in the first three zones, giving a total buffer of 30%. Since too many LLINs remained unused after issuing in these zones, the buffer stock at district level was subsequently decreased to 15%. Further, in order to reduce surplus buffer stocks at district level in the first five zones, remaining nets from these zones were re-distributed to the Coastal and Northern Zones, the last zones to receive nets.

#### LLIN ordering, production, inspection and delivery

After the compilation of district-level registration data, the logistics partner prepared a district packing list with the numbers of LLINs to be delivered to each district and village, including the calculated buffer stocks. The logistics contractor then sent the zonal order to NMCP for the approval of the MoHSW and NMCP forwarded the official purchase order to the LLIN supplier.

LLIN production was done by the local manufacturer, A-Z Textile Mills Ltd in Arusha, in several lots. The quality of each lot was inspected by an independent inspection company (Intertek International Ltd, Kenya). Most importantly, LLIN delivery to village level was the responsibility of the manufacturer. This was of enormous practical benefit to the programme as it took care of the most complex logistical problem, the distribution of the bulky nets to over 14,000 destinations throughout a country of 947,600 sq km with a limited road infrastructure [13].

When arriving at the recipient village, the supplier’s representative met with the VEO and the government storage facility keeper to deliver the agreed number of LLINs. Once the LLINs were delivered, the VEOs (DMOs in the case of the district buffer stocks) were responsible for storage until the LLINs were issued to beneficiaries.

### LLIN issuing

UCC issuing in each zone took place over three days (Friday to Sunday) to ensure a maximum number of eligible beneficiaries received an LLIN. Depending on its size, each village was divided into several predefined sectors and assigned one issuing point with two volunteers per sector to minimize travelling time to and waiting time at the issuing point. The VEO conducted training on issuing for volunteers and facilitated the storage and transportation of LLINs to the issuing points.

Beneficiaries brought their bar-coded coupons to the issuing point closest to their home and exchanged it for a LLIN. The thumbs of recipients were marked with an indelible ink to prevent them from claiming a second net and their thumbprint was also put on their coupons. The volunteers removed the barcode sticker from the net bag and placed it on the coupon to verify the transaction. Unregistered people, or people who had lost their coupons, were registered or re-registered by the VEO at the last day of issuing and given a LLIN from the buffer stock. Government officials from the NMCP participated in the issuing process by making courtesy calls at regional level and visiting selected issuing points for supervision. Donors also made periodic site visits to observe the issuing process.

After issuing was completed, the redeemed coupons were bundled and handed over to the VEO. The VEOs and WEOs then prepared respectively a Village and Ward Issuing Report. CHMTs collected the Ward Issuing Reports and paid allowances to the issuing volunteers and the local government officials. Reports and coupons were forwarded to the logistics contractor for compilation and scanning into the UCC LLIN database.

### Hang-up campaign

To ensure correct use and hanging of LLINs, the Tanzania Red Cross Society conducted a LLIN hang-up campaign in all rural districts about one week following LLIN issuing. Existing volunteers and community members who participated in the U5CC hang-up campaign were used in most cases. CHMTs were informed about the activity and regional and district stakeholders were trained to instruct district supervisors, who in turn trained the local supervisors and volunteers. Volunteers visited 50 to 70 households per day over five to seven days. In case the net had not been hung, volunteers assisted with hanging up the net. They also demonstrated the proper use of LLINs and advised households regarding net maintenance and the importance of consistent net use. Additionally, each household was provided a poster or sticker on proper and consistent LLIN use throughout the year.

### Social mobilization

The social mobilization contractor publicized the campaign before and during registration and issuing. This was done through mass media and community outreach activities, including television and radio spots, advertisements in the newspapers, rural film and cultural shows, brochures, posters, T-shirts as well as public meetings and announcements. Generally, the UCC mobilization efforts ensured that communities knew about the campaign plans and that the nets were free of charge to the beneficiaries. Also, the importance of sleeping under an ITN all year round was constantly emphasized.

### Monitoring and evaluation

Household surveys followed completion of the UCC and hang-up campaign in seven districts: two in the Southern zone (Nachingwea and Mtwara Urban), three in the Lake zone (Sengerema, Rorya and Chato) and two in the Coastal zone (Kisarawe and Rufij) (A–C, Figure 2). Surveys in the Southern zone were done in March and April 2011 (middle of the rainy season), in Lake zone in June 2011 (soon after the rainy season), and Coastal zone in October 2011 (dry season). While most of the previous household surveys were conducted in the dry season, evaluation of the UCC was done during different seasons, which can make it difficult to compare data between zones due to seasonality of net hanging. The objective of the surveys was to assess household ITN ownership and use for different age and risk groups. ITN use is defined as the percentage of a given population group that slept under an ITN the night before the survey.

A total of 887, 592 and 580 households were surveyed in the Lake, Southern and Coastal zones, respectively. The zones were selected in line with the sub-national NATNETS surveys for which provision had been made in the NMCP M&E Plan 2008–2013. Districts within a zone were chosen based on the availability of baseline data from the 2008 NATNETS national survey (Marchant T, Bruce J, Nathan R, Mponda H, Sedekia Y, Hanson K: Monitoring and Evaluation of the Tanzanian National Net Strategy, Report on 2008 NATNETS Household, Facility services and Facility users surveys, unpublished). Sampling at district level was done by selecting 10 clusters (villages) with the selection probability proportional to the size of the village. Within each village, one sub-village was chosen using simple random sampling. Afterwards, 30 households were chosen in each selected sub-village by using a modified EPI-type sampling procedure resulting in a total of 300 households per district. Design of the questionnaire was primarily guided by the U5CC household survey tool and focused on household ownership and use of ITNs among different risk groups (under-fives, pregnant women, all household members). As in the standard Malaria Indicator Survey questionnaires, an ITN was defined as: 1) a factory-treated net that does not require any further treatment (LLIN), or 2) any net that has been soaked with insecticide within the past 12 months [14]. Additional questions were added to capture several process indicators specific to the UCC (e g, awareness of the UCC or indicators related to the UCC registration and issuing procedure) (Nathan R, Sedekia Y: Monitoring and Evaluation of the Tanzanian National Net Strategy, Universal Coverage Campaign, Household Survey Report- Coastal zone, Lake and Southern zones, unpublished).

An equity ratio, defined as the value for the lowest wealth quintile divided by the value for the highest wealth quintile, was used to assess equity across socio-economic quintiles. Relative wealth was estimated as an index derived from a combination of the household head’s education, housing conditions, asset ownership of the household and whether the house was rented or not. Weights for the variables were derived using principal components analysis, leading to a continuous variable. Households were then divided into quintiles according to the value of their score, ranging from the poorest (quintile 1) to the least poor (quintile 5) [15].

### Sources of operational and financial data

Operational data were compiled from UCC final reports submitted to NMCP by the implementation partners. Further sources of operational data included: minutes of stakeholder meetings, e-mail exchanges, and internal NMCP documentation. For the financial data, all stakeholders were requested to indicate how much they contributed for the UCC per cost category, donor and grant (in the case of the GFATM). This information was cross-checked with the disbursements made by NMCP to contractors and a NATNETS expenditure overview prepared internally. Only direct financial expenses were compiled, without taking into account opportunity costs, the time spent by government officials and other indirect economic costs.

## Results

### Preparation

#### Coordination and planning

According to the original Round 8 proposal, UCC activities were supposed to start in February 2010 and be completed in October 2010. However, delayed grant signature, a lengthy process of tender document approval by GFATM and sub-recipient contracting delayed the UCC start until June 2010. Campaign activities were further disrupted in December 2010 when a revised Procurement and Supply Management plan was requested by GFATM to justify the increased requirement for nets exceeding the originally approved 14.6 million before their next disbursement could be made. As a result UCC issuing activities in the last two zones (Coastal and Northern) were put on hold between April and September 2011 pending the approval of the revised Procurement and Supply Management plan by GFATM and the associated interruption of the supplier’s manufacturing schedule. The timetable with final registration and issuing dates is given in Figure 3.

#### LLIN quantification and procurement

As had been observed during the U5CC, the Census data from 2002 and its projections for 2010 were not sufficiently reliable to determine accurate LLIN requirements for the UCC [11]. Thus, a significantly higher number of nets were needed compared with the original estimate. This discrepancy is shown in Table 3 on a zonal basis. Fortunately, as a result of the competitive tendering process the price quoted for LLINs (including delivery) from the most competitive bidder was lower than the budget for commodities (USD 4.39 *versus* USD 6.01), which provided financial room to procure the required number of nets. As another positive consequence of this low price, institutional sleeping spaces could also be covered in the campaign although no such provision had been made in the original grant proposal.

**Table 3:** **Estimated and registered sleeping spaces and number of long**-**lasting insecticidal nets** (**LLINs**) **delivered**, **issued and remaining**, **by zone on Tanzania mainland**

**Zone (3 admini-strative regions)**	Total sleeping spaces originally estimated*	**Household sleeping spaces registered**	**Institutional sleeping spaces registered†**	**Total sleeping spaces registered**	**LLINs delivered to village level**	**LLINs issued to households on official issuing days**
Southern	1,747,039	1,947,680	50,909	1,998,589	2,530,160	2,133,901
Southern Highlands	2,373,315	2,106,489	120,937	2,227,426	2,747,240	2,255,621
Central	2,226,024	2,004,892	170,070	2,174,962	2,605,880	2,194,885
West Lake	1,527,812	2,174,424	47,007	2,221,431	2,704,200	2,329,243
Lake	2,183,990	2,765,445	63,420	2,828,865	3,436,200	2,967,324
Coastal	2,763,931	2,714,578‡	88,592	2,803,170	2,717,840	3,004,487
Northern	1,787,480	1,708,945	95,676	1,804,621	1,462,520	1,736,790
**Total**	**14,609,591**	**15,422,453**	**636,611**	**16,059,064**	**18,204,040††**	**16,622,251**

	**LLINs issued to unmet sleeping spaces after official issuing days**	**LLINs issued to institutions**	**Total LLINs issued**	**LLINs issued (% of delivered)**	LLINs re-distributed from surplus buffer stocks**	**Total Number of LLINs still remaining within the zone (%)**
Southern	29,404	82,721	2,246,026	88.8	216,400	400 (0.02)
Southern Highlands	37,203	102,342	2,395,166	87.2	276,440	6,680 (0.24)
Central	120,424	136,392	2,451,701	94.1	122,160	11,400 (0.44)
West Lake	180,770	47,007	2,557,020	94.6	125,400	1,694 (0.06)
Lake	107,365	63,420	3,138,109	91.3	250,480	37,233 (1.08)
Coastal	0	88,592	3,093,079	113.8	0	310,521 (11.43) ‡‡
Northern	0	0	1,736,790	118.8	0	111,387 (7.62) ‡‡
**Total**	**475,166**	**520,474**	**17,617,891††**	**96**.**8**	**990,880**	**479,315 (2**.**63)††**

Source: MEDA Tanzania: Final Report Universal Coverage Campaign (unpublished document, 2012).

*National Bureau of Statistics estimates based on 2010 population projection from the 2002 census data.

†Institutional sleeping spaces (boarding schools, hospitals, prisons, military barracks, orphanages) were registered separately post (first three zones) or prior (last four zones) to the official issuing days.

‡This number does not include the number of sleeping spaces re-registered in Dar es Salaam in September 2011, where initially 1,381,124 sleeping spaces were registered and then an additional 203,445 spaces were re-registered and issued LLINs.

**615,000 LLINs from the first three zones were re-distributed to the Coastal zone and 375,880 LLINs from the last two zones to the Northern zone.

††The difference between the 18,204,040 LLINs delivered and the LLINs issued and still remaining within the zone (17,617,891 plus 479,315) equals 106,834 LLINs, which cannot be accounted for (0.59% of LLINs delivered).

‡‡A higher number of nets remain in these two zones because remaining nets following issuing days were not recollected as done in the other zones.

### Training

At district level, the training contractor organized a UCC introduction meeting for a total of 2,062 CHMT members, which corresponded to 107% of the original target. Additionally, 92% (486) of Division Secretaries, 96% (2,986) of WEOs and 98% (14,473) of VEOs were trained on how to carry out the UCC registration and issuing exercise (World Vision Tanzania: Universal Coverage Campaign, Final Implementation Report, unpublished).

### Registration of sleeping spaces

Registration of sleeping spaces was successfully completed countrywide by the end of February 2011 (Figure 3). A total of 16,059,064 sleeping spaces were registered, including 15,422,453 counted at 9,925,952 rural and urban households and an additional 636,611 found in institutions (Table 3).

In those zones sampled for post-campaign surveys (Lake, Southern and Central) 89.6%, 92.6% and 95.9% of the surveyed households were registered, respectively. Most of these were registered before the issuing days (99% in all zones).

### Delivery, issuing and buffer stock management

Table 3 shows that a total of 18,204,040 LLINs were procured and delivered to village level. Of these, 17,617,891 were issued in three ways: to households during official issuing days (16,622,251) or afterwards to unmet household sleeping spaces (475,166) or to institutions (520,474). As a result, 96.8% of the LLINs delivered to village level reached beneficiaries. In Coastal and Northern zones more than 100% of nets delivered by the supplier were issued because these zones also received redistributed surplus buffer stocks from the previous zones. Unissued LLINs did remain in all zones at the end of the campaign but different interventions, like buffer stock adjustments and LLIN redistribution, resulted in their numbers being reduced to less than 2.6% of the total LLINs delivered. Compared to other zones, a significantly higher number of LLINs remained in the Coastal and Northern zones (11.43% and 7.62% of LLINs delivered to village level) as in these two zones remaining nets following issuing days were not recollected as had been done in other zones. It has been recommended that these LLINs be issued to the new high school and college students in the coming semester.

In Lake, Southern and Coastal zones 86%, 92% and 95% of the households received at least one LLIN. Among households that received at least one net, 47% (Lake), 30% (Southern) and 50% (Coastal) received two nets and 14% (Lake), 41% (Southern) and 17% (Coastal) got three or more nets. Mean traveling time spent to get to the distribution point was 25, 20 and 16 minutes in Lake, Southern and Coastal Zones. Overall, 87%, 90% and 94% of the respondent in the Lake, Southern and Coastal Zones spent less than an hour to get to the issuing point. Thus, it can be concluded that distribution points were located fairly close to the residences (Nathan R, Sedekia Y: Monitoring and Evaluation of the Tanzanian National Net Strategy, Universal Coverage Campaign, Household Survey Report- Coastal zone, Lake and Southern zones, unpublished).

### Hang-up campaign

According to the routine data of the Tanzania Red Cross Society (TRCS), 25,191 volunteers visited 96% (6,912,456) of a total of 7,183,106 rural district households in mainland Tanzania. TRCS data from Northern and Coastal zone also show that in 87% of the households LLINs already hung at the time of the visit. In the same two zones volunteers assisted in hanging 47% of the nets that had not been hung before, resulting in five to eight nets a day if 50 to 70 households were visited. Assessed by the presence of the sticker that was to be delivered to the household by the hang-up campaign volunteers, household survey data collected by the monitoring and evaluation contractor showed that 44.9%, 43.9% and 47.2% of all the households that received at least one LLIN in Lake zone, Nachingwea District (only rural district within the Southern zone) and Coastal zone were visited (Nathan R, Sedekia Y: Monitoring and Evaluation of the Tanzanian National Net Strategy, Universal Coverage Campaign, Household Survey Report- Coastal zone, Lake and Southern zones, unpublished). However, these numbers may be underestimates as it is possible that households were visited, but stickers were not provided or removed later on.

### Net ownership and use following the UCC

In Tanzania mainland the national average of household ITN ownership was 17.9% in 2005 (Hanson K, Marchant T, Mponda H, Nathan R: Monitoring and Evaluation of the TNVS, Report on 2005 TNVS Household, Facility and Exit surveys, unpublished). In the households surveyed by the monitoring and evaluation contractor in Southern and Lake zones, ownership of at least one ITN per household in 2008 was 46.3% and 34.1%, respectively (Marchant T, Bruce J, Nathan R, Mponda H, Sedekia Y, Hanson K: Monitoring and Evaluation of the Tanzanian National Net Strategy, Report on 2008 NATNETS Household, Facility services and Facility users surveys, unpublished). Owing to the TNVS and the implementation of the U5CC, this indicator rose to 60.2% and 81.3% in 2010 and reached 96.0% and 95.2% post UCC in 2011. Coastal zone data were only collected for 2010 and 2011, but nevertheless an increase of 28 absolute percentage points from 69.8% to 97.8% could be observed within that time period (Table 4) (Nathan R, Sedekia Y: Monitoring and Evaluation of the Tanzanian National Net Strategy, Universal Coverage Campaign, Household Survey Report- Coastal zone, Lake and Southern zones, unpublished). Furthermore, preliminary results of the latest Tanzania HIV Malaria Indicator Survey (2011–12) show that net ownership reached 91.5% in mainland Tanzania, confirming that the national target of 90% household ITN ownership by 2013 [16] has been reached [17].

**Table 4:** **Household insecticide**-**treated net ownership and use among children under the age of five years**, **all household members and pregnant women for different geographic areas over time** (**2005**–**2012**) [17,24]

	**Southern zone**		**Lake zone**		**Coastal zone**		**Tanzania**	
**Year**	**N**	**% (95% CI)**	**N**	**% (95% CI)**	**N**	**% (95% CI)**	**N**	**% (95% CI)**
***Household ITN ownership***								
2005	NA	NA	NA	NA	NA	NA	6115	17.9 (15.7–20-2)
2006	NA	NA	NA	NA	NA	NA	6260	28.9 (26.6–31.3)
2007	NA	NA	NA	NA	NA	NA	6198	36 (33.1–38.9)
2008	875	46.3 (39.7–52.9)	1176	34.1 (28.2–40.5)	NA	NA	6922	45.7 (40.1–51.5)
2009	592	60.8 (56.8–64.6)	891	82 (79.4–84.5)	NA	NA	NA	NA
2010	591	60.2 (56.7–63.9)	889	81.3 (78.7–83.7)	589	69.8 (66.0–73.3)	NA	63.4†
2011	592	96 (94.3 – 97.4)	887	95.2 (93.6–96.4)	580	97.8 (96.2–98.7)	NA	NA
2012	NA	NA	NA	NA	NA	NA	NA	91.5*
***ITN Use***‡ ***Children*** <***5***††								
2005	NA	NA	NA	NA	NA	NA	5567	12.2 (10.2–14.4)
2006	NA	NA	NA	NA	NA	NA	5815	21.1 (18.8–23.7)
2007	NA	NA	NA	NA	NA	NA	6123	26.2 (23.5–29.0)
2008	638	25.9 (21.0–31.4)	1408	23.9 (19.1–29.4)	NA	NA	5701	28.8 (22.3–36.3)
2009	304	48 (42.3–53.8)	1184	62.2 (58.8–65.5)	NA	NA	7768	NA
2010	261	55.9 (50.1–61.6)	1047	71.3 (67.8–74.7)	387	77.5 (72.5–81.9)	NA	63.9†
2011	226	84.1 (77.9–88.7)	821	79.2 (75.8–82.2)	293	72.7 (66.8–77.9)	NA	NA
2012	NA	NA	NA	NA	NA	NA	NA	72.7*
***ITN Use***‡ ***All household members***								
2005	NA	NA	NA	NA	NA	NA	31164	9.8 (8.3–11.7)
2006	NA	NA	NA	NA	NA	NA	30273	15.4 (13.7–17.2)
2007	NA	NA	NA	NA	NA	NA	31381	20.5 (18.3–22.8)
2008	NA	18.8	NA	16.5	NA	NA	32246	25.8 (21.0–31.3)
2009	NA	29.9	NA	45.8	NA	NA	45125	NA
2010	NA	37.8	NA	56.6	NA	50.2	NA	45.1†
2011	2037	80.9 (78.5–83.0)	4561	69.6 (67.4–71.8)	2168	60.8 (57.4–64.1)	NA	NA
2012	NA	NA	NA	NA	NA	NA	NA	69.2*
***ITN Use***‡ ***Pregnant women***								
2005	NA	NA	NA	NA	NA	NA	779	10.7 (8.5–13.4)
2006	NA	NA	NA	NA	NA	NA	584	17.6 (14.2–21.7)
2007	NA	NA	NA	NA	NA	NA	707	23.2 (19.5–27.4)
2008	NA	24.2**	NA	20.1**	NA	NA	731	18.8 (12.9–26.5)
2009	NA	34.7**	NA	49.6**	NA	NA	922	NA
2010	NA	54.6	NA	63.9	NA	61.4	NA	57.1†
2011	514	82.1** (78.5–85.2)	695	75.8** (72.5–78.9)	395	65.1** (60.1–69.8)	NA	NA
2012	NA	NA	NA	NA	NA	NA	NA	76.2*

Source: Nathan R, Sedekia Y: Monitoring and Evaluation of the Tanzanian National Net Strategy, Universal Coverage Campaign, Household Survey Report- Coastal zone, Lake and Southern zones (unpublished document, 2011). *ITN* = insecticide-treated net; *NA* = Not available.

†Data from 2009–2010 Tanzania Demographic Health Survey.

*Preliminary results of the Tanzanian HIV and Malaria Indicator Survey 2011/12.

‡Percentage who slept under an ITN the past night.

††See Figure 5 for graphic representation of data.

**Data only available for women of child-bearing age (15–49).

ITN use of a given population group is shown in Table 4. ITN use of children under five years of age increased massively countrywide since 2005 (12.2%), reaching 72.7 % in 2012 according to the preliminary results of the latest Tanzania HIV Malaria Indicator Survey (Hanson K, Marchant T, Mponda H, Nathan R: Monitoring and Evaluation of the TNVS, Report on 2005 TNVS Household, Facility and Exit surveys, unpublished) [17]. Data from Southern and Lake zones for ITN use of children under five years of age demonstrate an improvement of 58.2 and 55.3 absolute percentage points in the last four years with values of 84.1% and 79.2% in 2011 after implementation of the UCC. Coastal zone values reached 72.7% in 2011. These usage levels confirm that Tanzania is well on track to reach its national target of 80% in the year 2013. Also, ITN use of all household members rose nationally from 9.8% in 2005 to 69.2% in 2012, while zonal values from Southern and Lake zones increased from 18.8% and 16.5% in 2008 to 37.8% and 56.6% in 2010 and 80.9% and 69.6% in 2011 post UCC. In Coastal zone an increase from 50.2% to 60.8% could be seen between 2010 and 2011, the years for which data was collected in this zone (Nathan R, Sedekia Y: Monitoring and Evaluation of the Tanzanian National Net Strategy, Universal Coverage Campaign, Household Survey Report- Coastal zone, Lake and Southern zones, unpublished).

### Equity following the UCC

**Figure 4:**
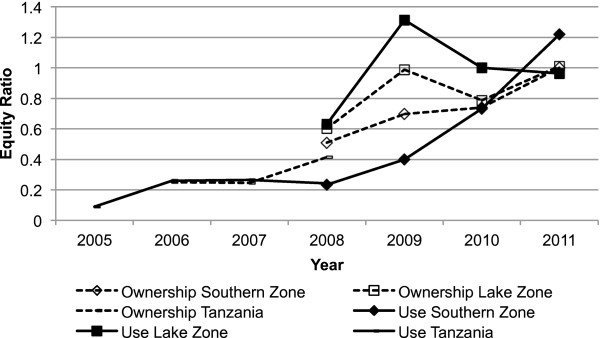
**Equity ratio of insecticide**-**treated net (ITN) household ownership and use in children under the age of five years, over time (2005**–**2011), mainland Tanzania.** ITN household ownership (dashed lines) and ITN use in children under the age of five years (solid lines) are shown for Tanzania mainland (triangles), Southern zone (diamonds) and Lake zone (squares) for the years 2005 to 2011. Coastal zone is not shown because there is only one data point (2011) available. Nationally representative data comes from national household surveys conducted by the monitoring and evaluation contractor. See main text for definition of equity ratios.

Estimates of household ITN ownership and ITN use by children under five years of age across socio-economic quintiles were used to assess equity. The equity ratio is depicted for both indicators over time (2005–2011), in Figure 4. Results from national surveys indicate a strong improvement in equity over time and the continuation of these trends was confirmed in the zones covered in the sub-national surveys since 2008. Thus gains in ITN ownership and use over the last seven years were higher in the lowest wealth quintiles than in the highest.

### Social mobilization

Based on project indicators of the social mobilization contractor all key deliverables were achieved or exceeded. For example, almost 50% more mobile video unit shows were conducted in rural areas than originally planned. The number of theatre shows, live radio programmes and posters were also above target. Press conferences were held, press releases issued, and prominent officials and personalities were invited for TV talk shows to ensure the campaign was widely publicized through print and electronic media. The only discrepancy between the planned and conducted activities was that more radio spots were placed in local radio stations and less in national stations, as during the campaign this approach was found to be more effective (Population Services International: Final Draft UCC Project Report, unpublished).

Awareness of the UCC among heads of households was very high, as shown by to the household surveys conducted after the campaign. Overall, 98.6%, 99.7% and 99.5% of the household heads in the Lake, Southern and Coastal Zones, had heard about the UCC. Radio was the most frequently cited source of information in the Lake (40%) and Southern (50%) zone, whereas in the Coastal Zone it was the community workers (37%). Additionally, the percentage of households registered before the issuing day was above 98% in all surveyed zones, supporting the fact that social mobilization efforts for the campaign were well received by a wide public (Nathan R, Sedekia Y: Monitoring and Evaluation of the Tanzanian National Net Strategy, Universal Coverage Campaign, Household Survey Report- Coastal zone, Lake and Southern zones, unpublished).

### Total campaign financing

The direct financial cost of the UCC is shown in Table 5, stratified by cost categories and contributions by donors. The total financial cost of the UCC was USD 96,402,293, resulting in a financial cost per LLIN delivered of USD 5.30 of which USD 4.39 represented the cost of the net (including delivery by the supplier) and USD 0.91 all other campaign costs. At 82.9% (USD 79,915,736) the cost category “LLIN supply and delivery” has the highest share of expenses relative to the total financial cost, followed by logistics with 8.81% (Table 5).

**Table 5:** **Financial cost** (**in un**-**adjusted United States Dollars** - **USD**) **of the Universal Coverage Campaign**, **by cost category and donor**

**Cost category**	**Donor**	**Amount in $**	Cost/LLIN* in $ (% of cost)
LLINs including delivery	Global Fund Round 8	79,915,736	4.39 (82.90)
LLINs redistribution	President’s Malaria Initiative	178,173	0.01 (0.18)
Logistics	Global Fund Round 8	5,758,203	
	Global Fund RCC	1,000,000†	
	President’s Malaria Initiative	1,736,000†	
Subtotal		8,494,203	0.47 (8.81)
Training	Global Fund Round 8	1,665,993	
	Global Fund RCC	1,015,540	
Subtotal		2,681,533	0.15 (2.78)
Social Mobilization	Global Fund Round 8	1,475,502	
	Global Fund RCC	75,895	
Subtotal		1,551,397	0.09 (1.61)
Hang-up Campaign	President’s Malaria Initiative	2,450,000	0.13 (2.54)
Process Audit	Global Fund Round 8	312,122	0.02 (0.32)
Monitoring and Evaluation	Global Fund RCC	178,787	0.01 (0.19)
Administration	Global Fund Round 8	378,248	
	SDC	262,096	
Subtotal		640,344	0.04 (0.66)
**Total**		**$96,402**,**293**	**$5.30** (**100**)

*LLIN* = long-lasting insecticidal nets; *RCC* = rolling continuation channel; *SDC* = Swiss Agency for Development and Cooperation.

*Cost category subtotal divided by the number of LLINs delivered (18,204,040).

†Estimates based on the disbursement made by NMCP and USAID.

‡During the time period of the UCC (14 months: June 2010 to April 2011 and August to October 2011) 40% of the NETCELL expenditures were accounted to UCC administration costs.

The main donor of the UCC was the GFATM with USD 91,776,025–USD 89,505,803 coming from the Round 8 grant and USD 2,270,222 from the Rolling Continuation Channel grant of Round 1. Apart from GFATM, PMI contributed USD 4,364,173 for redistributing the surplus buffer stocks, for logistics and for conducting the hang-up campaign.

## Conclusion

The close collaboration between the MoHSW, donors, contracted local partners, local government authorities and volunteers strongly contributed to the success of the UCC. The fact that local government stakeholders were the key implementers of the campaign activities had several advantages. Local leaders and community members felt a sense of pride for being recognized as suitable persons to implement the campaign (MEDA Tanzania: Final Report Universal Coverage Campaign, unpublished). Importantly, the use of local government administrative officials rather than healthcare workers placed no additional burden on the already understaffed Tanzanian healthcare system [18]. Additionally, it fostered the collaboration between government authorities at different levels as well as local partners, which contributed to strengthening the public sector generally. However, the campaign required extra time and high commitment from the public sector. There was also a need to pay allowances as the work was not considered an integral part of the official work and some local authorities even requested additional reimbursement for the accomplished work. Lack of evidence for the impact of the hang-up campaign and rather sparse data on the impact of social mobilization are the main limitation in the exercise and would require further investigations. Limitations in the original evaluation with only seven out of 125 districts being monitored and evaluated could be compensated with the availability of the preliminary results of the latest nationally representative Tanzania HIV Malaria Indicator Survey (2011–12).

With the delivery of 18.2 million LLINs, this campaign was one of the largest LLIN distribution efforts conducted to date in Africa. As of late 2012 only Nigeria and Democratic Republic of the Congo (DRC) have implemented campaigns of a larger size (46.9 million from 2009 to June 2012, and 19.1 million from 2011 to June 2012, respectively) (Alliance for Malaria Prevention: AMP country tracking by SRN, unpublished) [19]. Similar to Tanzania, Nigeria and DRC are using a rolling implementation mode on a state or province basis [20,21]. However, both countries receive considerable external operational support for campaign implementation [19-21], whereas in Tanzania most of the work was either done by the in-country LLIN manufacturer (transport logistics), local government entities, local NGO contractors or NMCP. For obvious reasons, being able to rely on existing systems that are not campaign-dependent has many advantages in terms of country ownership, operational sustainability and cost savings.

With an average cost of USD 5.30 per LLIN delivered, the Tanzanian UCC lies well below the median cost of delivery stated by the WHO in its latest World Malaria Report (USD 7.66) [4]. According to this report USD 5.30 is even less than the lowest price cited (USD 6.61) in studies conducted since 2005 [4]. This is supported by the review of White *et al*. on costs and cost-effectiveness of malaria control interventions, where the lowest financial cost per LLIN delivered was USD 6.01 [22]. Possible reasons for such a low price are (1) large size of this LLIN procurement, (2) local manufacturing and delivery capacity and (3) increased market competition with the number of WHOPES-recommended suppliers increasing from three in 2007 to 10 in 2011 [4]. In the case of the UCC, most of the expenses (83%) are accounted for by the cost of LLIN procurement and delivery. This compares favourably with average numbers mentioned by WHO according to which 70-85% of the cost is for LLIN procurement and 5-10% for LLIN delivery [4]. It is also comparable with the U5CC, where 80.4% of the total financial cost was used for procurement and delivery of LLINs [11].

Preliminary data from the THMIS collected after UCC completion indicate large improvements in ITN household ownership and use in all population groups. The results show that Tanzania is well on track to reach universal coverage, defined as at least 80% net usage, in the near future [1,17]. Further, it can be concluded that these achievements were reached in a fully equitable manner across wealth quintiles as demonstrated by an ownership and use equity ratio of 1 for all surveyed areas (Figure 4). Similar results were shown in a study done to evaluate the UCC by West *et al*. in Muleba District, north-west Tanzania, where they found an increase in ITN household ownership of at least one ITN (from 62.6% to 90.8%) and ITN use of children under five years of age (from 56.5% to 63.3%) post UCC as well as no association between net ownership and poverty [23].

**Figure 5:**
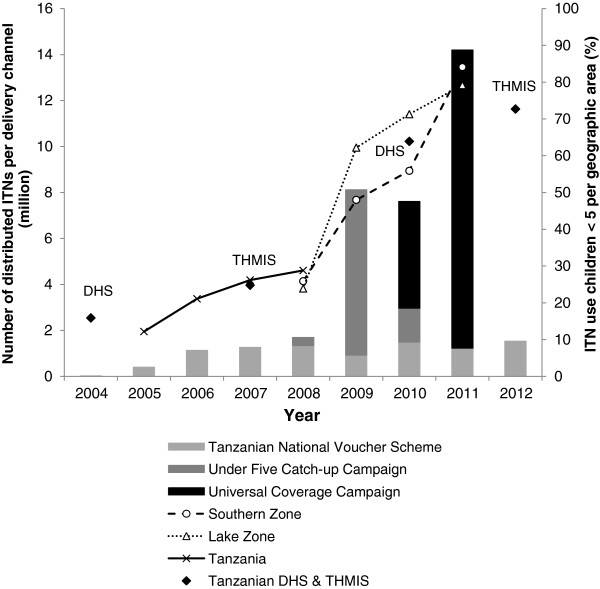
**Number of insecticide**-**treated nets (ITNs) distributed**, **per delivery channel and ITN use in children under the age of five years over time (2004–2012).** Number of ITNs distributed (bars) via the Tanzania National Voucher Scheme (light grey), Under-five Catch-up Campaign (medium grey) and Universal Coverage Campaign (dark grey) for the years 2004 to 2012. ITN use in children under the age of five years (lines and diamonds) is depicted for Southern zone (dashed line) and Lake zone (dotted line) as well as Tanzania mainland overall (solid line), including Demographic Health Survey (DHS) and Tanzania HIV and Malaria Indicator Survey (THMIS) data (diamonds). In order to retain readability of the figure, the available data points for Coastal zone (2010 and 2011) are not shown.

The outstanding progress made in these key indicators and the success of the UCC can be fully attributed to the activities of the NATNETS Programme and its partners. To scale-up ITN use in Tanzania mainland the NATNETS Programme invested from 2002 to 2011 approximately 300 million USD, out of which 57% came from the GFATM. Up to the end of 2011, the TNVS provided 7.8 million (22.7%) ITNs to pregnant women and infants. The U5CC delivered nationwide 9 million (26.2%) LLINs between 2008 and 2010 to children under five years of age. Thus, together with the 17.6 million (51.1%) LLIN issued through the UCC, a total of 34.5 million ITNs were distributed in Tanzania mainland from 2004 to 2011. As it can be seen in Figure 5, the increase in the number of ITNs delivered strongly correlates with the improvements in ITN use. The two campaigns conducted between 2008 and 2011 with a total financial cost of USD 160.2 million contributed massively to the steep increase in use. Regularly conducted nationally representative surveys (Demographic and Health Surveys and Tanzania HIV and Malaria Indicator Surveys) confirmed the sub-national data collected by the monitoring and evaluation contractor and provided national-level data [24-26].

In parallel to the massive increase in ITN use by all groups in the country, percentage of children under five years of age classified by rapid diagnostic test as having malaria decreased by 46% from 18.1% to 9.7% between 2007 and 2012 [17,26]. All-cause under-five child mortality fell by 45% between 1999 and 2010, from 147 deaths per 1,000 live births in 1999 to 81 per 1,000 live births in 2010 [27,28]. The progress and impact series of RBM about mainland Tanzania clearly states that even considering other factors that might explain the decline in all-cause under-five mortality, the role of malaria control in improving child survival is considerable. According to the LiST estimation model, which was used to estimate the number of lives saved among children under five in the same report, ITN scale-up efforts have averted more than 63,000 malaria deaths among children under five years of age in the past decade [27]. The MDG 4 target for Tanzania is to reduce the under-five mortality rate from 141 deaths per 1,000 live births in 1991 to a rate of 47 deaths per 1,000 live births in 2015 [29]. If the current decline seen between 1999 and 2010 continuous at the same rate, Tanzania will reach a rate of 51 deaths per 1,000 live births in 2015. Consequently, Tanzania will have almost achieved the MDG 4 (47 deaths per 1,000), as one of the few major African countries to do so.

However, to sustain this achievement in child mortality, malaria control efforts have to be maintained at the current high level. To do so, an ITN distribution strategy is urgently needed to maintain high coverage levels now that universal coverage has been reached (a so-called Keep-Up strategy [30,31]).

In the case of Tanzania mainland, one keep-up mechanism does fortunately already exist: the TNVS. At present the TNVS delivers around 1–1.2 million LLINs per year, less than 20% of the annual requirement of seven million LLINs to maintain universal coverage [32]. After extensive consultative deliberations, an additional strategy to directly deliver free LLINs through primary and secondary schools as a complement to the TNVS mechanism is believed to be the best way forward, considering operational feasibility, efficiency, yearly cost and impact [32]. Based on the calculations of the consultants, the combination of the TNVS and school net delivery will lead to a sustained coverage of about 82% use. If all households with possible access to nets through this combination were included, this strategy would cover 84% of all households representing 95% of the population [32]. The country is currently in the planning stages of piloting this new strategy. While Tanzania is well armed to successfully pursue malaria control activities, much of the success in the future will depend on (1) availability of international funding, and (2) increased domestic funding. National and international leadership is needed to ensure support is maintained for malaria control in the years ahead. Returning to an environment of holo-endemic malaria transmission across Tanzania cannot be considered an option.

## Abbreviations

CHMT: Council Health Management Team; DHS: Demographic and Health Survey; DMFP: District Malaria Focal Person; DMO: District Medical Officer; DRC: Democratic Republic of Congo; GFATM: Global Fund to Fight AIDS TB and Malaria; ITN: Insecticide Treated Net; LLIN: Long-Lasting Insecticidal Net; MDG: Millennium Development Goal; MEDA: Mennonite Economic Development Associates; MoHSW: Ministry of Health and Social Welfare; NATNETS: National Insecticide Treated Nets; NMCP: National Malaria Control Programme; PMI: United States President’s Malaria Initiative; RBM: Roll Back Malaria; RCC: Rolling Continuation Channel; SDC: Swiss Agency for Development and Cooperation; THMIS: Tanzania HIV and Malaria Indicator Survey; TNVS: Tanzanian National Voucher Scheme; TRCS: Tanzania Red Cross Society; U5CC: Under-five Catch-up Campaign; UCC: Universal Coverage Campaign; UCRC: Universal Coverage Registration Card; VEO: Village Executive Officer; WEO: Ward Executive Officer

## Competing interests

The authors declare that they have no competing interests.

## Authors’ contributions

CL contributed to the design of the methodology, coordinated the manuscript development and approval and participated in the editing process. RM strongly contributed to the design of the methodology and provided feedback on the manuscript. KK extensively participated in the analysis of the methodology and the editing process of the manuscript. FP, WR, AM, AM, RM, RM and JL made substantial contributions to the implementation and the analysis of the methodology. RN provided data on the results of the methodology and contributions to the manuscript. NJB participated in methodology design and implementation and substantially contributed to the development and revision of the manuscript. PM made contributions to the design of the methodology and the development and editing of the manuscript. SR participated in the implementation of the methodology and drafted the manuscript. All authors read and approved the final manuscript.
